# Comparative Analysis of Parasite Load on Recently Established Invasive Pumpkinseed *Lepomis gibbosus* (Actinopterygii: Centrarchidae) in Europe

**DOI:** 10.1007/s11686-024-00794-2

**Published:** 2024-03-01

**Authors:** Ivanna Dudliv, Yuriy Kvach, Maria Yu. Tkachenko, Kateryna Nazaruk, Markéta Ondračková

**Affiliations:** 1 Department of Zoology, Ivan Franko National University of Lviv, Hrushevskyi Str. 4, Lviv, 79005 Ukraine; 2 Institute of Vertebrate Biology of the Czech Academy of Sciences, Brno, Czech Republic; 3 Institute of Marine Biology, National Academy of Sciences of Ukraine, Odesa, Ukraine

**Keywords:** Co-introduction, Comparative analysis, Freshwater fish, Invasive alien species, Parasite communities, Parasitisation

## Abstract

**Purpose:**

The aim of this study was the comparative analysis of the parasite communities of new populations of invasive pumpkinseed (*Lepomis gibbosus*) in western Ukraine with pumpkinseed from Czechia, where populations have rapidly expanded over the last two decades.

**Methods:**

Sampling took place at three localities in the western part of Ukraine (i.e. Dobrotvir Reservoir (Vistula basin), Burshtyn Reservoir (Dniester basin), Mynai Pond (Danube basin)) and four in Czechia (i.e. Oxbow D2, Heršpický Pond (Danube basin), and Kolín oxbow and Římov Reservoir (Elbe basin).

**Results:**

In total, 11 parasite taxa were recorded in Ukraine and 17 in Czechia. Four species were co-introduced from North America with their host, i.e. the myxosporean *Myxobolus dechtiari*, the monogeneans *Onchocleidus dispar* and *Onchocleidus similis*, and metacercariae of a trematode *Posthodiplostomum centrarchi*. High dominance indices were related to a high abundance of co-introduced parasites, i.e. *O. similis* in Mynai pond and *P. centrarchi* in Dobrotvir Reservoir. Overall abundance of acquired parasites was generally low.

**Conclusion:**

This study shows that parasite communities in recently established pumpkinseed populations in the western part of Ukraine and Czechia are less diverse than those established in Europe for decades. The generally low parasite load in these new populations may play an important role in their ability to successfully establish and create strong populations by providing a competitive advantage over local species.

## Introduction

Parasites and pathogens are important components of natural ecosystems; their absence or presence affects interactions between a whole range of species in the community [1]. An increasing problem worldwide, however, is the presence of non-native parasites, which can negatively affect biota within their newly invaded ecosystems [2]. Many of these parasites are co-introduced together with their natural hosts [3, 4]. These parasites are mainly specific to their non-native hosts and remain part of their parasite fauna and do not infect local species during the host’s range expansion [5–7]. In some cases, however, the non-native host species may distribute new, non-native parasites, that successfully infect local hosts [8–10]. In such cases, the introduced non-native species may have a negative influence on local fauna as the new hosts lack the protective immunity that native hosts acquired during their shared coevolution history [11, 12].

Spread of parasites, pathogens and diseases is commonly associated with non-native fishes, and this can have serious consequences for local species as native hosts and non-native parasites have had no time to evolve an interaction balance [9, 13]. On the other hand, while local parasites can quickly assimilate new hosts, the temporary escape of parasites in the early stages of invasion allows non-native species to adapt to new environments and establish stable populations [14]. Subsequently, parasite infections of non-native host populations tend to increase over time after the onset of invasion, though infection rates rarely reach quantitative values similar to those of native species [15].

The pumpkinseed sunfish (*Lepomis gibbosus* L., 1758; Actinopterygii: Centrarchidae) is a North American freshwater species that was introduced into Europe at the end of the nineteenth century (1877) as an ornamental fish for garden ponds [16–18]. It is now distributed in at least 28 European states and in the Asian part of Turkey [19–22] and was included in the list of Invasive Species of Union Concern in 2019 [23]. In Ukraine, pumpkinseed was first recorded in 1918 in the Lower Danube [24], since when the fish has become common in the rivers and lakes of south Ukraine, the Dnipro (Dnieper) basin up to Kyiv, in small rivers of the Azov coast and in coastal waters of the Black Sea and the Sea of Azov [21, 25, 26]. In Czechia, the species first occurred in 1929 in the Třeboň region, where it was accidentally introduced with carp (*Cyprinus carpio* L., 1758) fry from the former Yugoslavia [27]. During the following years, just a few isolated populations were established [28]; however, rapid expansion into a wide range of water bodies has been observed over recent decades.

While there has been a relative scarcity of information available on parasites of pumpkinseed from its non-native range over the twentieth century (see e.g. [29, 30], and references in [31] for exceptions), research interest in this topic has increased over the last two decades. This is due, in part, to new data on co-introduced species such as myxozoans [32], monogeneans (e.g. [6, 33–35]), cestodes [36] and trematodes [37, 38]. In addition, several studies have now documented, or summarised, original data on parasite communities for populations from Western, Central and Eastern Europe. For example, pumpkinseed parasites are now well documented from Bulgaria [39], England [40], France [33, 36, 41, 42], Germany [43], Moldova [44, 45], Poland [30, 46], Slovakia and Serbia [31, 47]. However, despite the recent rapid expansion of pumpkinseed in both Czechia and Ukraine, data on their parasites are only known from a relatively small area of their host distribution. In Ukraine, pumpkinseed parasites have been studied in the Dnieper basin, Lower Danube and brackish water estuaries [29, 34, 48], while data from the western part of the country remains absent. Data from Czechia includes studies limited to a single parasite taxon, i.e. monogeneans, ciliates and crustaceans (e.g. [6, 49–51], though parasite community data have recently been published for a few isolated sites in the Danube and Elbe basins [42]. Thus, the aim of this study was to present data on the parasite communities of new pumpkinseed populations in the western part of Ukraine, and to these with data from Czechia, where pumpkinseed have rapidly expanded their distribution over the last two decades.

## Material and Methods

### Study Localities

**Fig. 1:**
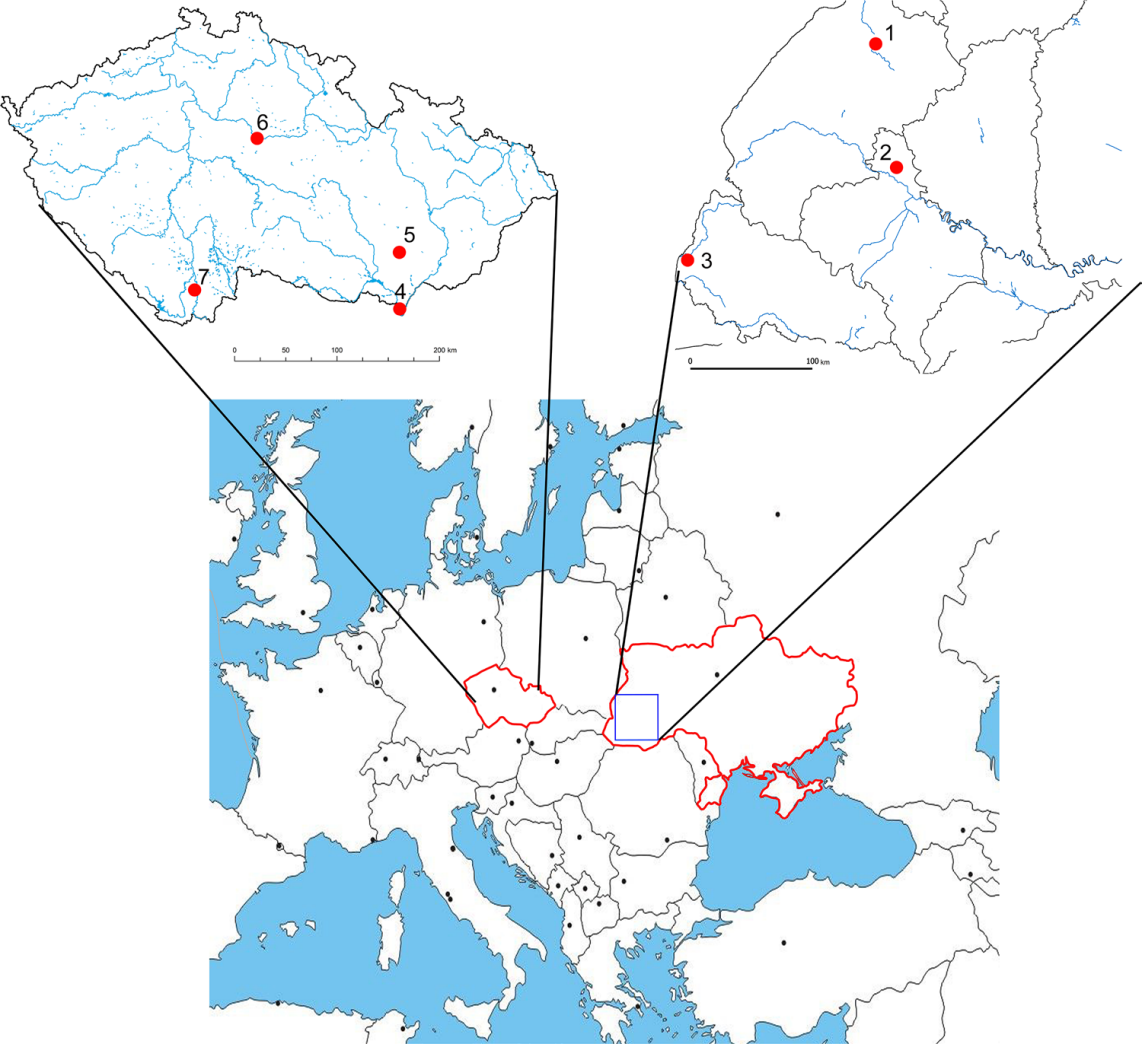
Schematic map with the study areas indicated. See “Material and methods” for the locality numbers

Sampling took place at three localities in the western part of Ukraine over June to September 2022, i.e. (1) Dobrotvir Reservoir (50.159799 N, 24.401295 E; Vistula basin); (2) Burshtyn Reservoir (49.257926 N, 24.639904 E; Dniester basin); and (3) Mynai Pond, Latorica River (48.58683N, 22.29009 E; Danube basin) (Fig. 1). In Czechia, fish were sampled from four waterbodies between 2015 and 2021, i.e. (4) oxbow D2 of the Dyje River (48.672910 N, 16.923282 E; Danube basin, 2015); (5) Heršpický Pond (49.114706 N, 16.932734 E; Danube basin, 2021); (6) Kolín oxbow (50.020453 N, 15.228106 E; Elbe basin, 2020); (7) Římov Reservoir, Malše River (48.835597 N, 14.482397 E; Elbe basin, 2018) (Fig. 1). The first locality, Dobrotvir Reservoir, is the only site in the Baltic basin and acts as a cooling reservoir for the Dobrotvir Power Plant on the Bug River (Vistula River tributary). The other two Ukrainian localities belong to the Black Sea basin. The Burshtyn Reservoir was built in 1965 on the Hnyla Lypa River (Dniester River tributary) as a cooling reservoir for the Burshtyn power plant but has now been reclassified as a natural protected area [52, 53]. The pond in the village of Mynai in the Transcarpathian region of Ukraine is a small fire pond located within the settlement and lacks any connection with a natural water body, though it lies within the Latorica River basin (Tisza River tributary, Danube River basin). In Czechia, both Oxbow D2 and Heršpický Pond lie within the Danube River basin. The first of these is an old oxbow in the lowest section of the Dyje River (Morava tributary) floodplain, created after the channelisation of the Dyje during the 1970s. Surrounded by trees and with a sand/mud bottom, almost 20% of the oxbow’s area is covered with aquatic vegetation. Pumpkinseed invaded Oxbow D2 sometime after 2010, since when they have established a large population [49]. Heršpický pond lies in the Svratka River basin and is now protected due to the occurrence and reproduction of several protected amphibian species. Pumpkinseed were accidentally introduced into the pond sometime around 2019, after which they quickly established a stable population (Zajíček R., personal observation). The final two Czech localities lie in the Elbe River basin (North Sea drainage). The Kolín oxbow was created after the Elbe River was channelised between 1913 and 1927 and is now recognised as a natural monument (Kolínské tůně) for protecting local ecosystems of the Elbe floodplain and their typical flora and fauna. Pumpkinseed was accidentally introduced into the oxbow sometime around the beginning of the twenty-first century. The canyon-shaped Římov Reservoir was constructed in 1978 by damming the River Malše (Vltava River tributary) for water supply and flood control. It is a 12 km long mesotrophic to eutrophic reservoir that lacks submerged aquatic macrophytes in the littoral zone due to its steep banks and water level fluctuations [54]. Occurrence of pumpkinseed in the reservoir has been known since 2008, the species probably entering the reservoir from the Malše River, into which it was introduced from adjacent fishponds (Kubečka J., personal observation).

### Fish sampling and Processing

All fish were collected using an angular 4 mm mesh trap-net (tetrahedron, 0.8 m edge), a 7 m, 1 cm mesh seine net or through backpack electrofishing (Lena, Bednář, Czech Republic). The fish were transported alive in aerated containers with water from the sampling locality to the laboratory, where they were subsequently held in aerated aquaria. Parasitological examinations were performed within 24 h after sampling, according to Kvach *et al.* [55].

A total of 127 fish were sampled and examined, comprising 52 specimens from Ukraine and 75 from Czechia (Table 1), with 22 fish collected from the Baltic basin, 70 from the Black Sea basin (12 in the Dniester and 58 in the Danube River drainages), and 32 from the North Sea basin. The standard (SL, mm) and total (TL, mm) lengths of each fish were measured before sectioning.

**Table 1:** Number and size of fish sampled from different localities in Ukraine and Czechia

Parameters	Ukraine			Czechia			
	Dobrotvir Reservoir	Burshtyn Reservoir	Mynai Pond	Oxbow D2	Heršpický Pond	Kolín oxbow	Římov Reservoir
No.	22	12	18	20	20	18	17
SL, mm							
*m* ± sd	71.6 ± 16.5	75.4 ± 21.7	68.2 ± 7.0	64.8 ± 3.6	73.5 ± 6.4	73.0 ± 27.9	77.4 ± 15.0
Min–max	8.0–96.0	51.0–117.0	60.0–82.0	70.2–90.2	75.0–101.0	47.2–136.0	44.0–121.1
TL, mm							
*m* ± sd	91.9 ± 9.3	93.4 ± 25.0	82.1 ± 7.7	79.8 ± 4.4	89.1 ± 8.2	89.2 ± 33.2	94.2 ± 17.8
Min–max	77.0–114.0	60.0–143.0	71.0–101.0	56.1–73.4	62.0–83.0	37.5–111.6	35.6–101.0

### Parasite Analysis

Fish tissues and organs were examined for the presence of parasites using Konus Crystal 7x-45x (Konus Optical and Sport Systems, Italy) and Olympus SZX 10 (Olympus Optical Co., Tokyo, Japan) stereomicroscopes. The fins, skin and gills were all observed in a Petri dish with water from the sampling locality, while the eyes, muscles and internal organs were examined after compression between two 9 × 13-cm glass plates. Microparasites were studied alive, monogeneans were preserved in GAP (Glycerine-ammonium-picrate) and prepared as semi-permanent slides [56], and digeneans, cestodes and nematodes preserved in hot 4% formaldehyde and stained with iron acetic carmine, dehydrated in ethanol of increasing concentration and mounted in Canada balsam as permanent slides [57, 58]. Acanthocephalans were preserved in 70% ethanol, pressed between two glass slides and then mounted in glycerol as temporary slides for light microscopy, while glochidia and crustaceans were preserved in 4% formaldehyde. All parasites were identified to species level where possible, or to the lowest possible taxon, under an Olympus IX 83 light microscope (Olympus Optical Co., Tokyo, Japan). Images of *Posthodiplostomum centrarchi* Hoffman, 1958 were obtained using an Olympus BX53 light microscope (Olympus Optical Co., Tokyo, Japan) fitted with the Olympus cellSens Standard v.3.2 digital image analysis package (Olympus Optical Co., Hamburg, Germany).

Indices of prevalence (P, %), mean intensity (MI), intensity range (IR) and mean abundance (A) were calculated for each parasite species [59], with standard deviation (sd) calculated for mean parameters. Similarity between parasite communities at different localities was evaluated using the Czekanowski-Sørensen Index (CSI) [60]. Differences in parasite abundance and infracommunity richness (i.e. number of parasite species per host) [59] between different pumpkinseed populations were tested using the Kruskal–Wallis ANOVA function in Statistica v.14.0.0.15 (TIBCO Software Inc., USA). Shannon Wiener, Dominance and Equitability diversity indices were calculated using PAST software (PAlaeontologicalSTatistics, v.1.77, http://folk.uio.no/ohammer/past/) [61] for populations with two or more parasite taxa. Only metazoan parasites were used for diversity indices as the intensity of infection was not possible to calculate for protozoan parasites. The same software was also used for permutation tests to compare diversity indices between the same locality. Bonferroni correction for multiple comparisons was used, with the level of significance set at *p* < 0.01.

### Molecular Analysis

Parasite specimens were preserved in 96% ethanol for the identification of white grub metacercariae from internal organs. DNA was extracted from each metacercaria using the innuPREP Forensic Kit (Analytik Jena, Germany). PCR was undertaken using the internal transcribed spacer 1 (ITS1) specific primers BD1 (forward: 5′-GTCGTAACAAGGTTTCCGTA-3′) and 4S (reverse: 5′-TCTAGATGCGTTCGAARTGTCGATG-3′) [62, 63]. The PCR reaction mix had a total volume of 10 μl, comprising 4 μl of extracted DNA, 0.3 μl primer, 2 μl buffer A, 0.2 μl dNTPs, 0.2 μl MgCl_2_, 0.1 μl Taq polymerase and 2.9 μl ddH2O. The KAPA2G Robust Hot-Star PCR Kit (Kapabiosystems, USA) was used for PCR analysis. Amplification was undertaken using a Mastercycler ep gradient S thermocycler (Eppendorf, Germany) with an annealing temperature of 57 °C. The PCR product was then purified using an ExoSAP-IT kit (Affymetrix Inc., Santa Clara, USA), according to the manufacturer’s protocol. The PCR products were then checked on 1.5% agarose gel with GoodView™ Nucleic Acid Stain. Sanger sequencing of the PCR products was performed commercially at GATC Biotech (Germany) and the sequences were edited and aligned using Geneious v.9.0.5 software [64].

The obtained sequences were compared with the NCBI database using BLASTn to assess sequence similarity with *Posthodiplostomum* sp. 3 (GenBank accession no. HM064955), *Posthodiplostomum* sp. 5 (HM064958) and *Posthodiplostomum* sp. 8 (HM064962) [65], and with *Posthodiplostomum* cf. *minimum centrarchi* (KX931441, MF170981-82, MF170992-93) [37].

## Results

### Pumpkinseed Parasite Community Composition

In total, 25 parasite taxa were recorded on pumpkinseed from the two study regions (11 taxa in Ukraine, and 17 in Czechia) (Table 2). In Ukraine, the richest parasite community, consisting of seven taxa, occurred in the Dobrotvir Reservoir. Only one species was found in the Burshtyn Reservoir and four in the Mynai Pond. In Czechia, the richest parasite community was found in the Kolín and D2 oxbows (nine and seven taxa, respectively; Table 3). Most of the localities compared had no common parasite species. In the other cases, similarity was less than 50%, being 25% between the Burshtyn and Dobrotvir reservoirs, 23.5% between the D2 and Kolín oxbows, 22.2% between D2 and Římov, 16.7% between Kolín and Římov and 14.3% between D2 and Dobrotvir. Only two species occurred in both Ukraine and Czechia, the North American monogenean *Onchocleidus dispar* Mueller, 1936 and the trematode metecarcariae *P. centrarchi* (ICS = 14.3%).

**Table 2:** Mean parasite abundance in pumpkinseed from different localities in Ukraine and Czechia

#	Parasite species	Site	Ukraine			Czechia			
			Dobrotvir Reservoir	Burshtyn Reservoir	Mynai Pond	Oxbow D2	Heršpický Pond	Kolín oxbow	Římov Reservoir
Ciliata									
1	*Trichodina* spp.	Fins				2.8		11.7	
2	*Oodinium* spp.	Fins						1.2	
Myxozoa									
3	*Myxobolus dechtiari* Cone & Anderson, 1977	Gills			0.1				
4	Myxozoa gen. sp.	Swim bladder, mesentery	0.2	0.1					
Monogenea									
5	*Onchocleidus dispar* Müller, 1936	Gills			0.4	0.4			
6	*Onchocleidus similis* Müller, 1936	Gills			15.2				
Cestoda									
7	*Triaenophorus nodulosus* (Pallas, 1781) (plerocercoids)	Mesentery					0.1		
8	*Bothriocestus claviceps* (Goeze, 1782)	Gut						0.1	
	*B. claviceps* (procercoids)	Gut						0.2	
9	*Valipora* spp. (plerocercoids)	Gall bladder						0.2	
Digenea									
10	*Posthodiplostomum centrarchi* Hoffman, 1958 (metacercariae)	Internal organs, muscles, gills	13.0			6.8			
11	Echinochasmidae gen. sp. (metacercariae)	Gills				0.9			
12	*Holostephanus dubinini* Vojtek & Vojtkova, 1968 (metacercariae)	Liver	0.05						
Acanthocephala									
13	*Pomphorhynchus laevis* (Zoega in Müller, 1776) (larva)	Liver	0.05						
14	*Acanthocephalus lucii* (Müller, 1776)	Gut							0.4
Nematoda									
15	*Eustrongylides excisus* Jägerskiöld, 1909 (larvae)	Liver, mesenterium	0.1						
16	*Schulmanela petruschewskii* (Schulman, 1948)	Liver				0.1			
17	*Raphidascaris acus* (Bloch, 1779) (larvae)	Gut						0.1	
18	*Anguillicola crassus* Kuwahara *et al.*, 1974 (larvae)	Gonads, gut wall						0.4	
Bivalvia									
19	*Sinanodonta woodiana* (I. Lea, 1834) (glochidia)	Fins, gills, gill covers				0.8			
20	*Unionidae* gen. sp. (glochidia)	Gills	0.3						
Crustacea									
21	*Neoergasilus japonicus* (Harada, 1930)	Fins						0.1	
22	*Ergasilus sieboldi* von Nordmann, 1832	Gills						0.1	
23	*Lernaea cyprinacea* L., 1758	Fins	0.05						
24	*Argulus foliaceus* (L., 1758)	Gills, fins				0.1		0.1	0.3
Arachnida									
25	Unionicolidae gen.sp.	Gills			0.1				
	Richness		7	1	4	7	1	9	2

**Table 3:** Pumpkinseed parasite diversity at different localities in Ukraine and Czechia

Locality	Basin	Species richness	Diversity index		
			Shannon	Dominance D	Equitability J
Ukraine					
Dobrotvir Reservoir	Bug/Vistula	7	0.290	0.897	0.149
Burshtyn Reservoir	Dniester	1	1	0	
Mynai pond	Tisza/Danube	4	0.175	0.932	0.126
Czech Republic					
Oxbow D2	Dyje/Danube	7	0.883	0.575	0.493
Heršpický Pond	Svratka/Danube	1	1	0	
Kolín oxbow	Elbe	9	1.777	0.197	0.913
Římov Reservoir	Malse/Elbe	2	0.679	0.514	0.980

**Fig. 2:**
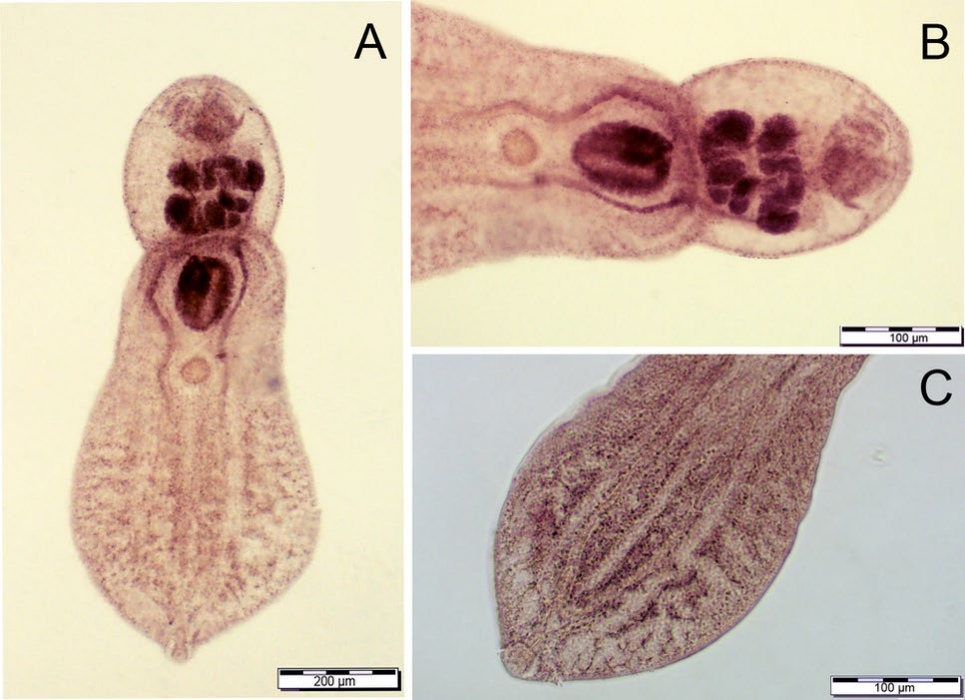
*Posthodiplostomum centrarchi ex Lepomis gibbosus* metacercaria from the Dobrotvir Reservoir. **A** Ventral view; **B** posterior end (gonads primordia and brandes visible); **C** anterior view

**Fig. 3:**
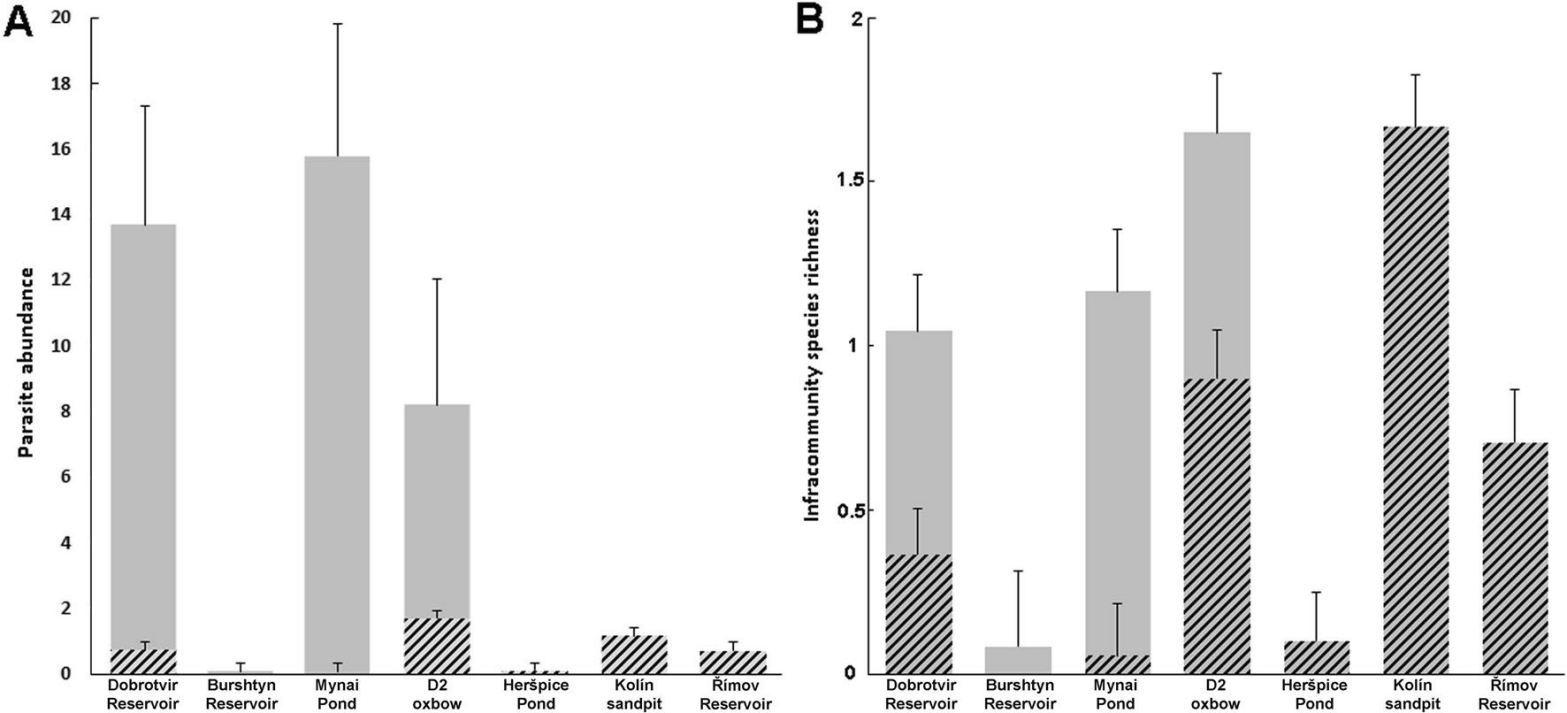
**A** Parasite abundance (without microparasites), and **B** infracommunity species richness in *Lepomis gibbosus* collected from different localities in Ukraine and the Czech Republic (bars = mean, whiskers = S.E.). Grey bars = values for all parasite taxa, within which the hatched parts represent parasites acquired in Europe

We found four species co-introduced from North America along with their host, the myxosporean *Myxobolus dechtiari* Cone & Anderson, 1977 (Mynai pond, Burshtyn Reservoir), the monogeneans *O. dispar* (Mynai pond, Oxbow D2) and *Onchocleidus similis* Mueller, 1936 (Mynai pond) and metacercariae of the trematode *P. centrarchi* (Dobrotvir Reservoir, Oxbow D2). Identification of *P. centrarchi* was confirmed through genetic analysis, with two sequences obtained from the Dobrotvir Reservoir and one from Oxbow D2. One of the sequences from the Dobrotvir Reservoir and the D2 oxbow were identical and differed from the second sequence from the Dobrotvir Reservoir with one substitution and one gap. All sequences showed 99.9% correspondence with *P. centrarchi* from pumpkinseed collected in the Hudson River in Canada (GeneBank Acc. No. MH521251), Hungary (MN080282) and in Portugal (MF171006; denoted as *P*. cf. *minimum*) Morphological characteristics were—body length: 1467.4 ± 190.0 (1171.4–1673.6), forebody length: 996.6 ± 93.2 (855.2–1154.59) and width: 495.2 ± 79.5 (377.4–617.4), hindbody length 536.9 ± 75.3 (406.4–635.1) and width 476.5 ± 93.6 (350.9–630.2), oral sucker length 45.5 ± 5.7 (36.9–55.6) and width 47.3 ± 5.8 (41.2–57.9), ventral sucker length 56.6 ± 7.7 (45.1–70.1) and width 55.6 ± 5.3 (49.8–63.9) and Brandes organ length 166.9 ± 20.0 (136.1–196.2) and width 164.9 ± 33.9 (121.5–229.9) (Fig. 2). Where present, the North-American parasites reached highest prevalence and abundance, representing 89% of all parasites collected (excluding ciliates, for which abundance was not calculated; Fig. 3A). No co-introduced North-American parasites were observed in the Burshtyn Reservoir (Ukraine), Heršpický Pond, Kolín oxbow or in Římov Reservoir (Czechia) (Table 2, Fig. 3).

Regarding parasites acquired in Europe, all except two taxa each occurred at one locality only, with generally low abundance (Fig. 3A). The branchiuran *Argulus foliaceus* (Linnaeus, 1758) was the most frequent species found at the three localities in Czechia, though all infected fish were parasitised with a single specimen only. Trichodinid ectoparasites were observed at two localities in Czechia, with 56% of fish infected at the Kolín oxbow and just one fish infected at Oxbow D (Table 2). In addition to local species, pumpkinseed collected in Czechia acquired three Asian species, glochidia of invasive Chinese pond mussels (*Sinanodonta woodiana* (I. Lea, 1834)) in Oxbow D2, and ergasilid copepods *Neoergasilus japonicus* Harada, 1930 and larvae of the eel (*Anguilla anguilla* (L., 1758)) nematode *Anguillicola crassus* Kuwahara, Niimi & Itagaki, 1974 at the Kolín oxbow. In comparison, parasite acquisition in Ukrainian regions was rare, with most parasites found being of North American origin (see above).

### Comparative Analysis of Parasite Abundance

Parasite abundance differed significantly between localities (Kruskal–Wallis ANOVA; H (6, *N* = 127) = 66.7, *p* < 0.0001; Fig. 3A), with just one or two species recorded at three localities (Burshtyn, Heršpický pond and Římov Reservoir) and a mean abundance of greater than five at three others (Mynai, Dobrotvir and Oxbow D2; Table 2). Interestingly, these latter three sites were the only ones where co-introduced North American parasites were found. Regarding parasites acquired in Europe, i.e. excluding North American parasites, differences in parasite abundance between localities diminished and, while overall analysis showed significant differences (H (6, *N* = 127) = 34.9, *p* < 0.0001), post-hoc comparison tests indicated no significant difference between localities after correction for multiple comparisons.

### Parasite Diversity

Diversity varied greatly among sites, both in Czechia and Ukraine (Table 3). Infracommunity species richness was highest in the Kolín and D2 oxbows, and lowest in the Burshtyn and Heršpický ponds (Fig. 3B). Component community species richness ranged from one in both regions (Burshtyn, Heršpický pond) to seven in Ukraine (Dobrotvir) and nine in Czechia (Kolín). Analysis of localities with two or more parasite taxa indicated that Ukrainian localities were characterised by a low Shannon diversity index and equitability, alongside high dominance, this being related to a high abundance of co-introduced parasites, i.e. the monogenean *O. similis* in the Mynai pond and the trematode *P. centrarchi* in the Dobrotvir Reservoir (Tables 2, 3). On the other hand, we observed a high Shannon index, but rather low dominance and equitability at Czech localities (Table 3). The maximum Shannon index, recorded at the Kolín oxbow (1.78), was significantly higher than that at Mynai, Dobrotvir, Římov and Oxbow D2 (permutation test, *p* < 0.001). In addition, Shannon diversity at Mynai was significantly lower than that at Oxbow D2 (p < 0.001). Dominance indices at Mynai and Dobrotvir were significantly higher than those at all other Czech localities (all p ≤ 0.002), while dominance at Kolín was lower than that at Oxbow D2 and Římov. Equitability was significantly higher at Czech localities compared with Ukrainian localities and was also significantly lower at Oxbow D2 compared with other Czech localities (*p* < 0.001 for all comparisons).

## Discussion

We compared the parasite communities of new invasive pumpkinseed populations in the western part of Ukraine with pumpkinseed from Czechia, where populations have rapidly expanded over the last two decades. In total, 11 parasite taxa were recorded in Ukraine and 17 in Czechia, with four species having been co-introduced from North America with their host. Metacercariae of *P. centrarchi* were registered in pumpkinseed from Ukraine for the first time, and we also provide the first report of the *Posthodiplostomum* sp. 3 lineage sensu Locke *et al.* [65]. Previously, *P. centrarchi* metacercariae had been found in largemouth bass *Micropterus salmoides* (Lacépède, 1802) in Ukraine, having been transported from France and held in aquacultural ponds in Kyiv [35]. These trematodes are related to the *Posthodiplostomum* sp. 8 lineage, which appears to be specific to largemouth bass [35, 65]. Over the last decade, metacercariae of *P. centrarchi* have been recorded in several European countries, including Bulgaria, Czechia, France, Germany, Hungary, Poland, Portugal and Slovakia [37, 38, 42, 43, 46, 66], with most belonging to the *Posthodiplostomum* sp. 3 lineage collected from pumpkinseed. Though a recent study of pumpkinseed populations in southern Ukraine failed to confirm the presence of this parasite species in the region [48], further expansion of *P. centrarchi* to the east is to be expected. The definitive hosts of this parasite are herons (Ardeidae) [38, 67], which spread the eggs of the parasites to new localities. The presence of established populations of the second intermediate host in Ukraine, i.e. the pumpkinseed, makes possible the completion of the parasite’s life cycle.

Among the other co-introduced parasites, the myxozoan *M. dechtiari* has already been recorded in Ukraine in Lake Kartal and the brackish Khadzhibey Estuary [48]. This parasite is currently known in invasive pumpkinseed in Hungary, Moldova and Portugal [32, 44]. Considering the recent finding of this parasite in the Tisza River basin in Ukraine, close to the Hungarian border, and in the Lower Danube basin (Lake Kartal), it is plausible that the range of this species is much wider in the Danubian basin.

Two co-introduced monogenean species*, O. dispar* and *O. similis*, are widely distributed in Europe, having been recorded in non-native pumpkinseed populations in Austria, Bulgaria, Croatia, Czechia, France, Germany, Italy, Norway, Serbia and Slovakia [6, 33, 42, 43, 47, 68, 69]. In Ukraine, both species are known from the Danube basin [70], but only *O. dispar* has been found in the Dnipro (Dnieper) river basin [34, 48]. In Czechia, while both *Onchocleidus* species have previously been recorded from pumpkinseed caught in the Dyje River in the Danube River basin and the Donbas sandpit in the Morava River basin [6, 42], recent pumpkinseed populations in waterbodies of the Dyje River floodplain appear to be parasitised with *O. dispar* only [this study, 42]. These results suggest that the pumpkinseed making up the most recent invasion of lentic waterbodies of the Dyje River floodplain do not originate from long-established Dyje pumpkinseed populations, which are parasitised with both *O. similis* and *O. dispar* [6]. This study also confirmed the presence of *O. dispar* and *O. similis* in the Latorica river basin in the Transcarpathian region of Ukraine. Pumpkinseed were first recorded in the Tisza basin in the 2000s, since when the species has started to establish large populations in many adjacent waterbodies [71, 72]. The presence of these non-native monogeneans strongly suggests that these populations are related to the old Danubian pumpkinseed populations.

The low parasite load observed in most of the localities in Czechia and the western part of Ukraine examined in this study could be explained by the young age of the pumpkinseed populations. Low parasite prevalence was particularly apparent in relation to species acquired in Europe. The occurrence of pumpkinseed in the Bug River basin (Dobrotvir Reservoir) is new, and our finding is, in fact, the first record of this fish species in the Vistula River basin, the species only having been recorded in the Oder River basin in the Baltic Sea catchment prior to this study [73, 74]. Despite being a recent population, pumpkinseed parasite species richness in the Dobrotvir Reservoir was relatively high, reaching the same level as that found in other Ukrainian localities [48]. However, most parasite species found only occurred accidentally, with the North American trematodes *P. centrarchi* predominating, resulting in the high dominance index recorded at this locality. The same situation was observed at Oxbow D2 on the Dyje River in Czechia, where the population was approximately three to four years old. Fish here had acquired seven parasite taxa, though at very low abundance and prevalence, with *P. centrarchi* representing the majority of parasites in the community.

Extreme parasite avoidance was observed at Burshtyn Reservoir and Heršpický Pond, where only one and two parasites were found, respectively. The Burshtyn Reservoir (Upper Dniester River basin) is another new locality for pumpkinseed in Ukraine and represents the furthest upstream population reported to date from the Dnieseter basin, the species having been previously recorded in the middle reach and the delta and estuary [75–77]. The population in the Heršpický pond in Czechia is also a recent invasion but of unknown origin. As noted above, the parasite community was extremely poor at both sites, which corresponds with the ‘parasite-loss theory’ of Torchin *et al.* [78]. While fish in the Upper Dniester were only infected by a single unidentified myxozoan found in the swim-bladder, larvae of the cestode *Triaenophorus nodulosus* (Pallas, 1781) were also found rarely in fish from the Heršpický pond (see Table 2). Like Burshtyn Reservoir and Heršpický Pond, the parasite community at Římov Reservoir comprised just two local species (*Acanthocephalus lucii* Müller, 1776, *Argulus foliaceus*), though at higher prevalence than the other sites. These data tend to agree with the ‘enemy release hypothesis’, which explains the success of early invasions through temporary release from parasites and pathogens [78–80].

Parasite acquisition by pumpkinseed in both regions studied was generally low, particularly at localities with recently established pumpkinseed populations, which agrees with findings from other European water bodies. In comparison, the older, established population in the Kolín oxbow, which was introduced ca. 15 years before this investigation, was characterised by a high parasite diversity and equitability, low dominance and highest species richness at both component and infracommunity levels. Similar examples of temporal acquisition of local parasites have been described for other non-native fish species, e.g. round goby (*Neogobius melanostomus* (Pallas, 1814)) [15, 81]. Our data, showing high diversity and equitability at the Kolín oxbow, therefore, indicate that 15 years after population establishment, the parasite community reached certain stability, although being composed of acquired parasites only. The component community species richness of nine spp. was only slightly lower compared to old-established populations in Germany and France, where 10–11 spp. were found using the same parasite collection methodology and sample size [42].

The species richness of co-introduced North American parasites was also generally low when compared to older European populations in France and Germany [42]. For example, five co-introduced parasite species, *Actinocleidus oculatus* (Mueller, 1934), *Actinocleidus recurvatus* Mizelle & Donahue, 1944, *O. dispar*, *O. similis* and *P. centrarchi*, showed high abundance in the Rhine basin in Germany [43], while the same species were observed in France accompanied by *Cleidodiscus robustus* Mueller, 1934, *Gyrodactylus macrochiri* Hoffman & Putz, 1964 and *Proteocephalus ambloplitis* (Leidy, 1887)[33, 36]. Pumpkinseed from these regions, which represent the first area of European pumpkinseed introduction [82], exhibit high genetic diversity alongside their high North American parasite diversity [42]. Thus, the low parasite load of co-introduced parasites in more recent populations in Czechia and the western part of Ukraine may be indicative of ‘dilution’ from the source pumpkinseed populations.

This study showed that parasite communities in recently established pumpkinseed populations in Ukraine and Czechia are less diverse than those established decades previously and that this applies to both co-introduced and acquired parasite species. The generally low parasite loads in these new populations may play an important role in their ability to successfully establish and create strong populations and may provide a competitive advantage over local species.
